# Supplementary material for: Yeast eIF2A has a minimal role in translation initiation and uORF-mediated translational control in vivo
Source: bioRxiv. 2023 Dec 9:2023.10.06.561292. Originally published 2023 Oct 8. Preprint. [Version 2] doi: 10.1101/2023.10.06.561292 (PMC10659434; doi:10.1101/2023.10.06.561292)
Supplement: Supplement 1 [file NIHPP2023.10.06.561292V2-supplement-1.pdf]

bioRxiv preprint doi: https://doi.org/10.1101/2023.10.06.561292; this version posted December 9, 2023. The copyright holder for this preprint (which was not certified by peer review) is the author/funder. This article is a US Government work. It is not subject to copyright under 17 USC 105 and is also made available for use under a CC0 license.

SUPPLEMENTARY FIGURE LEGENDS

Figure 1-figure supplement 1. High reproducibility between biological replicates of ribosome footprint profiling and RNA-seq analyses.

(A-H) Scatterplots depict the RPF (A, C, E, G) or mRNA (B, D, F, H) read densities for all expressed mRNAs across biological replicates of the WT (A, B), the *eIF2AΔ* mutant (C, D) SM-treated WT (E, F) and SM-treated *eIF2AΔ* mutant (G, H). The read densities were calculated by mapping the reads to the CDS of each gene and expressed as reads per million mapped reads (RPM) in individual libraries of biological replicates. The Pearson's coefficient (r) is indicated in each plot, quantifying the degree of correlation between the replicate datasets.

Figure 2-figure supplement 1. Relative TE changes evoked by increased eIF2α phosphorylation in cells lacking eIF2A are broadly similar to relative TE changes conferred by increased eIF2α phosphorylation in WT cells.

(A) Volcano plot as in Figure 2A showing the $\log_2$ ratios of TEs in SM-treated *eIF2A*Δ versus untreated *eIF2AΔ* cells ($\Delta TE_{eIF2A\Delta+SM/eIF2A\Delta}$ values) for the 5426 mRNAs with evidence of translation. The dotted line marks the 1% FDR threshold. Genes showing a significant increase ($\Delta TE_{eIF2A\Delta+SM/eIF2A\Delta}$_up) or decrease ($\Delta TE_{eIF2A\Delta+SM/eIF2A\Delta}$_down) in TE in SM-treated *eIF2AΔ* versus *eIF2AΔ* mutant cells at FDR < 0.05, are plotted in dark and light blue circles, respectively. **(B)** Proportional Venn diagram showing overlap between the 1884 mRNAs identified in Figure 2B and the 786 mRNAs identified in Figure 2-figure supplement 1A.

Figure 3-figure supplement 1. Representative separation of polysomes by sedimentation through a sucrose density gradient in the experiment depicted in Figure 3E.

bioRxiv preprint doi: https://doi.org/10.1101/2023.10.06.561292; this version posted December 9, 2023. The copyright holder for this preprint (which was not certified by peer review) is the author/funder. This article is a US Government work. It is not subject to copyright under 17 USC 105 and is also made available for use under a CC0 license.

The A_{260} values were determined continuously during fractionation of the gradient. Fractions pooled for isolation of RNA from 80S monosomes or the various polysomal species are indicated by boxes.

Figure 6-figure supplement 1. eIF2A plays a minimal in regulating uORF-mediated translation.

(A-B) Smoothed scatterplots displaying the relationship between $\log_2 RRO_{WT}$ (x-axis) and $\log_2 RRO_{eIF2A\Delta}$ (y-axis) for all mRNAs containing annotated AUG- or NCC-uORFs (A) or evolutionarily conserved AUG- or NCC-uORFs (B) in WT versus *eIF2AΔ* cells without SM treatment. No mRNAs showed ≥2-fold changes in RRO in the *eIF2AΔ* mutant versus WT cells at FDR < 0.5. **(C-D)** Smoothed scatterplots displaying the relationship between $\log_2 RRO_{WT+SM}$ (x-axis) versus $\log_2 RRO_{eIF2A\Delta+SM}$ (y-axis) for the same mRNAs analyzed in (A)-(B) but in the presence of SM. Again, no mRNAs showed ≥2-fold changes in RRO in the *eIF2AΔ* mutant versus WT cells at FDR < 0.5. **(E-H)** Notched box plot displaying $\log_2$RRO values for all mRNAs containing annotated AUG- or NCC-uORFs (E, G) or evolutionarily conserved AUG- or NCC-uORFs (F, H) in untreated WT and *eIF2AΔ* mutant (E-F) or SM-treated WT and *eIF2AΔ* mutant (G-H). The y-axis scale was expanded by omitting a few outliers. Statistical significance determined using the Mann-Whitney U test is shown for the bracketed comparisons in panels E & G.

Figure 6-figure supplement 2. Lack of genetic interaction between eIF2A and the purine salvage pathway.

(A-B) Cell spotting assays were performed on SC plates (A) or SD (B) plates to assess the growth of WT, *eIF2AΔ*, *fcy2Δ* and *eIF2AΔ fcy2Δ* strains. Ten-fold serial dilutions of saturated cultures were applied to SC or SD plates supplemented with the indicated concentrations of adenine and incubated at 30°C for 2 days. **(C)** WT and *eIF2AΔ* strains were transformed with the indicated *lacZ* reporter plasmids- IMD2 and IMD3. The transformants bearing the reporter plasmids-IMD2 containing IMD2 coding sequences along with 1186 bp upstream region while IMD3 contains IMD3 coding sequences containing 555 bp upstream region- were grown in SC-Ura to saturation. The cultures were then diluted in fresh SC-Ura containing 0.015 mM

bioRxiv preprint doi: https://doi.org/10.1101/2023.10.06.561292; this version posted December 9, 2023. The copyright holder for this preprint (which was not certified by peer review) is the author/funder. This article is a US Government work. It is not subject to copyright under 17 USC 105 and is also made available for use under a CC0 license.

concentration of Adenine and grown for 6 h to A_{600} of ~1.0. WCEs were prepared and assayed for β-galactosidase activities in units of nmol of ONPG cleaved per mg of protein per min. The results represent the fold change of means and ±SEMs of activities calculated from three independent transformants.

bioRxiv preprint doi: https://doi.org/10.1101/2023.10.06.561292; this version posted December 9, 2023. The copyright holder for this preprint (which was not certified by peer review) is the author/funder. This article is a US Government work. It is not subject to copyright under 17 USC 105 and is also made available for use under a CC0 license.

FIGURE SOURCE DATA

Figure 2-source data 1.

Spreadsheet tabulates the $\log_2$ ratios of TEs in *eIF2AΔ* versus WT cells ($\log_2\Delta TE_{eIF2A\Delta/WT}$ values) for each mRNA and the corresponding FDR determined by DESeq2 analysis of ribosome profiling and parallel RNA Seq data for the 5340 mRNAs with evidence of translation (Figure 2A).

Figure 2-source data 2.

Spreadsheet tabulates the $\log_2$ ratios of TEs in WT+SM cells versus WT cells ($\log_2\Delta TE_{WT+SM/WT}$ values) and the corresponding FDR determined by DESeq2 analysis of ribosome profiling and parallel RNA Seq data for the 5441 mRNAs with evidence of translation (Figure 2B & Figure 2-figure supplement 1B).

Figure 2-source data 3.

Spreadsheet tabulates the $\log_2$ ratios $\log_2\Delta TE$ values for the indicated mutant/condition for the indicated mRNA groups identified in Figure 2B (Figure 2D).

Figure 2-figure supplement 1-source data 1.

Spreadsheet tabulates the $\log_2$ ratios of TEs in SM-treated *eIF2AΔ* versus untreated *eIF2AΔ* cells ($\Delta TE_{eIF2A\Delta+SM/eIF2A\Delta}$ values) for each mRNA and the corresponding FDR determined by DESeq2 analysis of ribosome profiling and parallel RNA Seq data for the 5426 mRNAs with evidence of translation (Figure 2-figure supplement 1).

Figure 3-source data 1.

Spreadsheet tabulates the $\log_2$ ratios of TEs in *eIF2AΔ* cells treated with SM versus WT cells treated with SM ($\log_2\Delta TE_{eIF2A\Delta+SM/WT+SM}$ values) for each mRNA and the corresponding FDR determined by DESeq2 analysis of ribosome profiling and parallel RNA Seq data for the 5482 mRNAs with evidence of translation (Figure 3A).

Figure 3-source data 2.

bioRxiv preprint doi: https://doi.org/10.1101/2023.10.06.561292; this version posted December 9, 2023. The copyright holder for this preprint (which was not certified by peer review) is the author/funder. This article is a US Government work. It is not subject to copyright under 17 USC 105 and is also made available for use under a CC0 license.

Spreadsheet tabulates the $\log_2\Delta$TE values for the indicated mutant/condition for the 32 mRNAs in the group $\Delta TE_{eIF2A\Delta\text{+SM/WT+SM\_down}}$ defined in Figure 3A (Figure 3B).

Figure 3-source data 3.

Spreadsheet tabulates the $\log_2\Delta$TE values for the indicated mutant/condition for the 32 mRNAs in the group $\Delta TE_{eIF2A\Delta\text{+SM/WT+SM\_down}}$ defined in Figure 3A (Figure 3C).

Figure 3-source data 4.

Spreadsheet tabulates the changes in luciferase activity expressed from F.LUC reporters calculated for WT+SM versus WT and *eIF2AΔ+SM* versus *eIF2AΔ* for three biological replicates (Figure 3D).

Figure 3-source data 5.

Spreadsheet 1, "raw CT values for mRNA from qRT", tabulates the raw CT values for mRNA from qRT reactions for three biological replicates-a, b, and c. Spreadsheet 2, "Dilution factor exemplar WT_a", provides an example file for one of the biological replicate WT (wild type) samples, illustrating how to calculate the dilution factor. Spreadsheet 3, "% 18S rRNA", provides an example file illustrating how to calculate the dilution factor. Spreadsheet 4, "Area % & Polysome norm factor", tabulates following parameters for all the biological replicates: Area under peaks from UV trace; SUM 80S+Polysomes; Average SUM 80S+Polysomes; Polysome recovery normalisation factor; and % total Area under 80S + Polysomes from UV trace in each peak. Spreadsheet 5, "rRNA normalisation factor", illustrates the calculations required to determine the rRNA normalization factor. Spreadsheet 6, "SAG1", illustrates the calculations required to determine ΔMonosome-Polysome/Total RNA (See materials and methods section for details) (Figure 3E).

Figure 6-source data 1.

Spreadsheet 1-3, "Fig 6A(i-iii)", tabulates the lists and $\log_2\Delta$TE values for the indicated mutant/condition for the annotated AUG- or NCC-uORFs (Fig 6Ai), conserved AUG- or NCC-uORFs (Fig 6Aii), or single functional inhibitory AUG-uORFs (Fig 6Aiii) (Figure 6A).

Figure 6-source data 2.

bioRxiv preprint doi: https://doi.org/10.1101/2023.10.06.561292; this version posted December 9, 2023. The copyright holder for this preprint (which was not certified by peer review) is the author/funder. This article is a US Government work. It is not subject to copyright under 17 USC 105 and is also made available for use under a CC0 license.

Spreadsheet 1-3, "Fig 6B(i-iii)", tabulates the lists and $\log_2\Delta$TE values as in (Figure 6-source data 1) for the subsets of the same mRNA groups analyzed there exhibiting > 1.41-fold increases in TE in SM-treated versus untreated WT cells (Figure 6B).

Figure 6-source data 3.

Spreadsheet 1-3, "Fig 6C(i-iii)", tabulates the lists and $\log_2\Delta$TE values as in (Figure 6-source data 1) for the subsets of the same mRNA groups analyzed there exhibiting > 1.41-fold decreases in TE in SM-treated *eIF2AΔ* versus SM-treated WT cells (Figure 6C).

Figure 6-source data 4.

Spreadsheet tabulates lists of the 514 mRNAs bearing functional AUG or NCC-uORFs, 17 mRNAs identified in Figure 3A showing evidence for a conditional requirement for eIF2A when eIF2 function is reduced by SM i.e., $\Delta TE_{eIF2A\Delta+SM/WT+SM}$_down* (N=17) group, and overlap of mRNAs with functional AUG- or NCC-uORFs (N=514) and $\Delta TE_{eIF2A\Delta+SM/WT+SM}$_down (N=17) (Figure 6D).

Figure 6-source data 5.

Spreadsheet tabulates the list of annotated AUG- or NCC-uORFs, chromosome coordinates, start codon of uORF, distances of the uORF AUG from the 5' end of the mRNA and the main CDS start codon, and the gene name.

Figure 6-source data 6.

Spreadsheet tabulates the list of conserved AUG- or NCC-uORFs, chromosome coordinates, start codon of uORF, distances of the uORF AUG from the 5' end of the mRNA and the main CDS start codon, and the gene name.

Figure 6-source data 7.

Spreadsheet tabulates the functional uORFs , chromosome coordinates, start codon of uORF, distances of the uORF AUG from the 5' end of the mRNA and the main CDS start codon, and the gene name (May et al. 2023).

Figure 6-figure supplement 1-source data 1.

bioRxiv preprint doi: https://doi.org/10.1101/2023.10.06.561292; this version posted December 9, 2023. The copyright holder for this preprint (which was not certified by peer review) is the author/funder. This article is a US Government work. It is not subject to copyright under 17 USC 105 and is also made available for use under a CC0 license.

Spreadsheet 1 tabulates the $\log_2$ ratios of following parameters for all the expressed annotated uAUG or NCC-uORFs listed in col. A in *eIF2AΔ* versus WT cells: Relative Ribosome Occupancy (RRO) in *eIF2AΔ* versus WT cells (RRO Change), RRO for WT, and RRO of *eIF2AΔ* (Figure 6-figure supplement 1A & E).

Figure 6-figure supplement 1-source data 2.

Spreadsheet 1 tabulates the $\log_2$ ratios of following parameters for all the evolutionarily conserved expressed uAUG or NCC-uORFs listed in col. A in *eIF2AΔ* versus WT cells: Relative Ribosome Occupancy (RRO) in *eIF2AΔ* versus WT cells (RRO Change), RRO for WT, and RRO of *eIF2AΔ* (Figure 6-figure supplement 1B & F).

Figure 6-figure supplement 1-source data 3.

Spreadsheet 1 tabulates the $\log_2$ ratios of following parameters for all the expressed annotated uAUG or NCC-uORFs listed in col. A in SM-treated WT and *eIF2AΔ* mutant: Relative Ribosome Occupancy (RRO) in *eIF2AΔ* treated with SM versus WT cells treated with SM (RRO Change), RRO for WT treated with SM, and RRO of *eIF2AΔ* treated with SM (Figure 6-figure supplement 1C & G).

Figure 6-figure supplement 1-source data 4.

Spreadsheet 1 tabulates the $\log_2$ ratios of following parameters for all the evolutionarily conserved expressed uAUG or NCC-uORFs listed in col. A in *eIF2AΔ* treated with SM versus WT cells treated with SM: Relative Ribosome Occupancy (RRO) in SM-treated WT and *eIF2AΔ* mutant (RRO Change), RRO for WT treated with SM, and RRO of *eIF2AΔ* treated with SM (Figure 6-figure supplement 1D & H).

Figure 6-figure supplement 1-source data 5.

Spreadsheet 1 tabulates the β-galactosidase activities in units of nmol of ONPG cleaved per mg of protein per min in WT and *eIF2AΔ* strains. Additionally, the spreadsheet provides the fold change of means and the associated ±SEMs (Standard Error of the Means) of these activities. These values have been calculated based on data obtained from three independent transformants for each condition (Figure 6-figure supplement 2B).

[illegible]
(which was not certified by peer review) is the author/funder. This article is a US Government work. It is not subject to copyright under 17 U
105 and is also made available for use under a CC0 license.

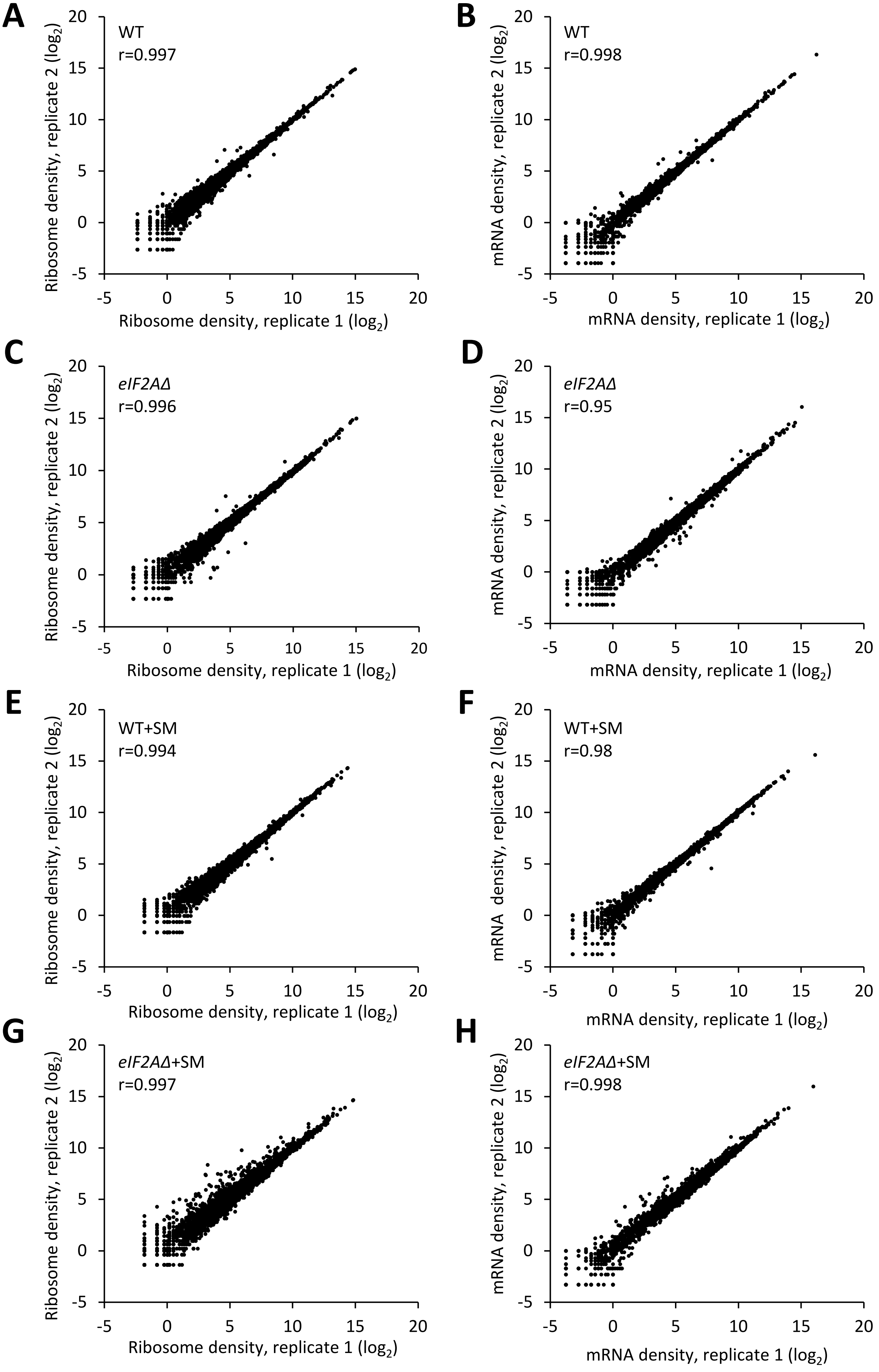

Figure 1-figure supplement 1

(which was not certified by peer review) is the author/funder. This article is a US Government work. It is not subject to copyright under 17 U
105 and is also made available for use under a CC0 license.

Figure 1-figure supplement 1. High reproducibility between biological replicates of ribosome footprint profiling and RNA-seq analyses.

(A-H) Scatterplots depict the RPF (A, C, E, G) or mRNA (B, D, F, H) read densities for all expressed mRNAs across biological replicates of the WT (A, B), the *eIF2AΔ* mutant (C, D) SM-treated WT (E, F) and SM-treated *eIF2AΔ* mutant (G, H). The read densities were calculated by mapping the reads to the CDS of each gene and expressed as reads per million mapped reads (RPM) in individual libraries of biological replicates. The Pearson's coefficient (r) is indicated in each plot, quantifying the degree of correlation between the replicate datasets.

(which was not certified by peer review) is the author/funder. This article is a US Government work. It is not subject to copyright under 17 U
105 and is also made available for use under a CC0 license.

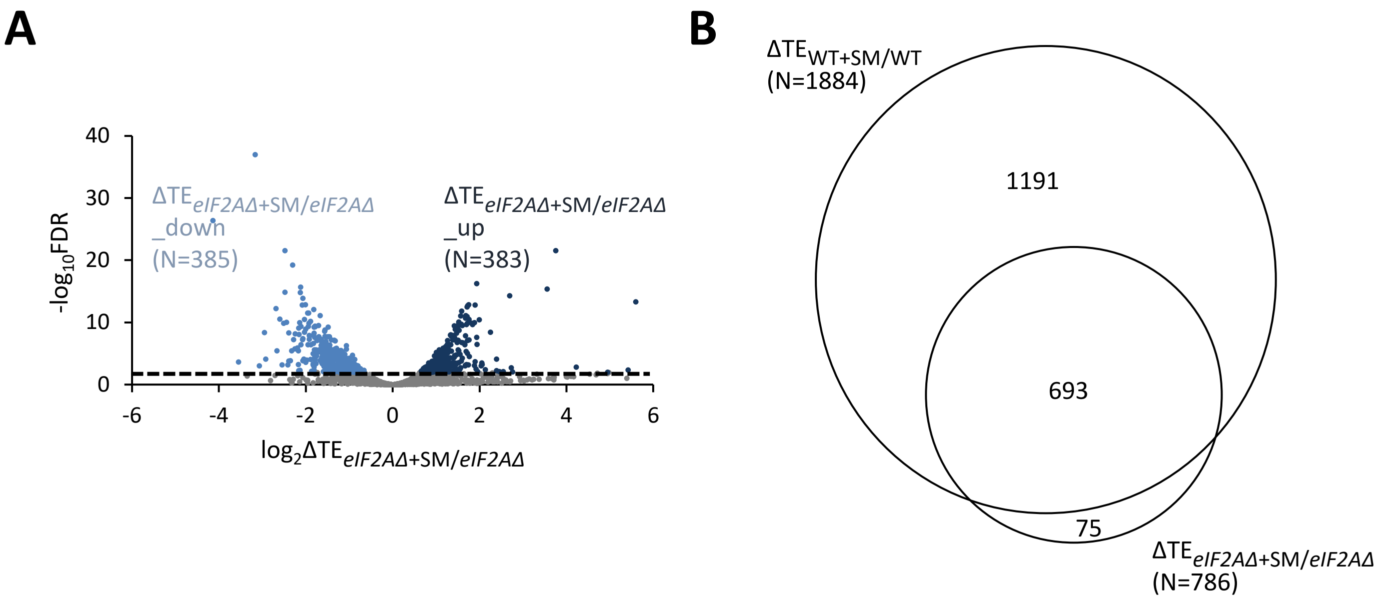

Figure 2-figure supplement 1. Relative TE changes evoked by increased eIF2α phosphorylation in cells lacking eIF2A are broadly similar to relative TE changes conferred by increased eIF2α phosphorylation in WT cells.

(A) Volcano plot as in Figure 2A showing the $\log_2$ ratios of TEs in SM-treated *eIF2A*Δ versus untreated *eIF2AΔ* cells ($\Delta TE_{eIF2A\Delta+SM/eIF2A\Delta}$ values) for the 5426 mRNAs with evidence of translation. The dotted line marks the 1% FDR threshold. Genes showing a significant increase ($\Delta TE_{eIF2A\Delta+SM/eIF2A\Delta}$_up) or decrease ($\Delta TE_{eIF2A\Delta+SM/eIF2A\Delta}$_down) in TE in SM-treated *eIF2AΔ* versus *eIF2AΔ* mutant cells at FDR < 0.05, are plotted in dark and light blue circles, respectively. **(B)** Proportional Venn diagram showing overlap between the 1884 mRNAs identified in Figure 2B and the 786 mRNAs identified in Figure 2-figure supplement 1A.

Figure 2-figure supplement 1

[illegible]
(which was not certified by peer review) is the author/funder. This article is a US Government work. It is not subject to copyright under 17 U
105 and is also made available for use under a CC0 license.

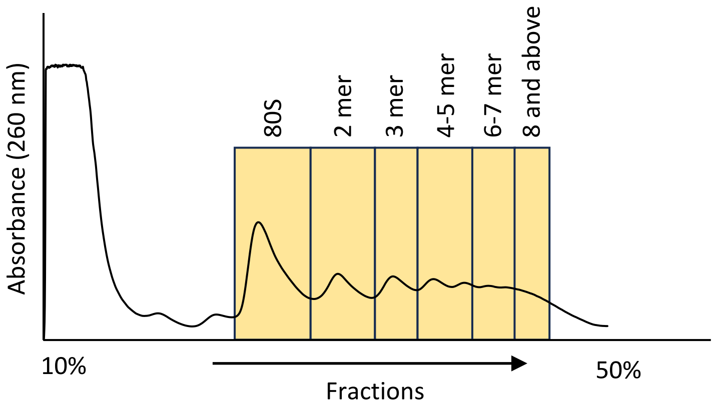

Figure 3-figure supplement 1. Representative separation of polysomes by sedimentation through a sucrose density gradient in the experiment depicted in Figure 3E.

The A_{260} values were determined continuously during fractionation of the gradient. Fractions pooled for isolation of RNA from 80S monosomes or the various polysomal species are indicated by boxes.

Figure 3-figure supplement 1

(which was not certified by peer review) is the author/funder. This article is a US Government work. It is not subject to copyright under 17 U
105 and is also made available for use under a CC0 license.

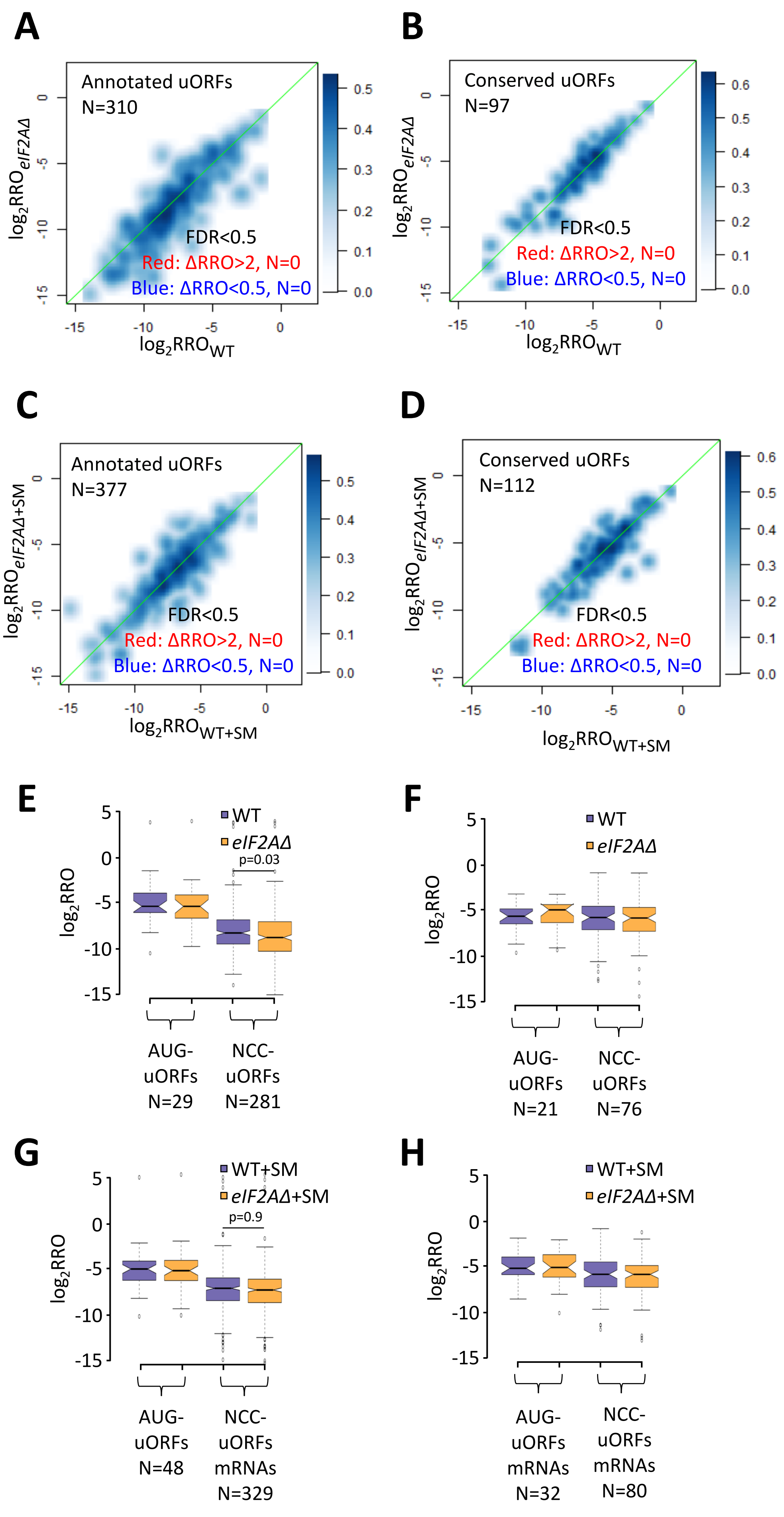

Figure 6-figure supplement 1

(which was not certified by peer review) is the author/funder. This article is a US Government work. It is not subject to copyright under 17 U
105 and is also made available for use under a CC0 license.

Figure 6-figure supplement 1. eIF2A plays a minimal in regulating uORF-mediated translation.

(A-B) Smoothed scatterplots displaying the relationship between log_2RRO_{WT} (x-axis) and $log_2RRO_{eIF2A\Delta}$ (y-axis) for all mRNAs containing annotated AUG- or NCC-uORFs (A) or evolutionarily conserved AUG- or NCC-uORFs (B) in WT versus *eIF2AΔ* cells without SM treatment. No mRNAs showed ≥2-fold changes in RRO in the *eIF2AΔ* mutant versus WT cells at FDR < 0.5. **(C-D)** Smoothed scatterplots displaying the relationship between log_2RRO_{WT+SM} (x-axis) versus $log_2RRO_{eIF2A\Delta+SM}$ (y-axis) for the same mRNAs analyzed in (A)-(B) but in the presence of SM. Again, no mRNAs showed ≥2-fold changes in RRO in the *eIF2AΔ* mutant versus WT cells at FDR < 0.5. **(E-H)** Notched box plot displaying log_2RRO values for all mRNAs containing annotated AUG- or NCC-uORFs (E, G) or evolutionarily conserved AUG- or NCC-uORFs (F, H) in untreated WT and *eIF2AΔ* mutant (E-F) or SM-treated WT and *eIF2AΔ* mutant (G-H). The y-axis scale was expanded by omitting a few outliers. Statistical significance determined using the Mann-Whitney U test is shown for the bracketed comparisons in panels E & G.

(which was not certified by peer review) is the author/funder. This article is a US Government work. It is not subject to copyright under 17 U
105 and is also made available for use under a CC0 license.

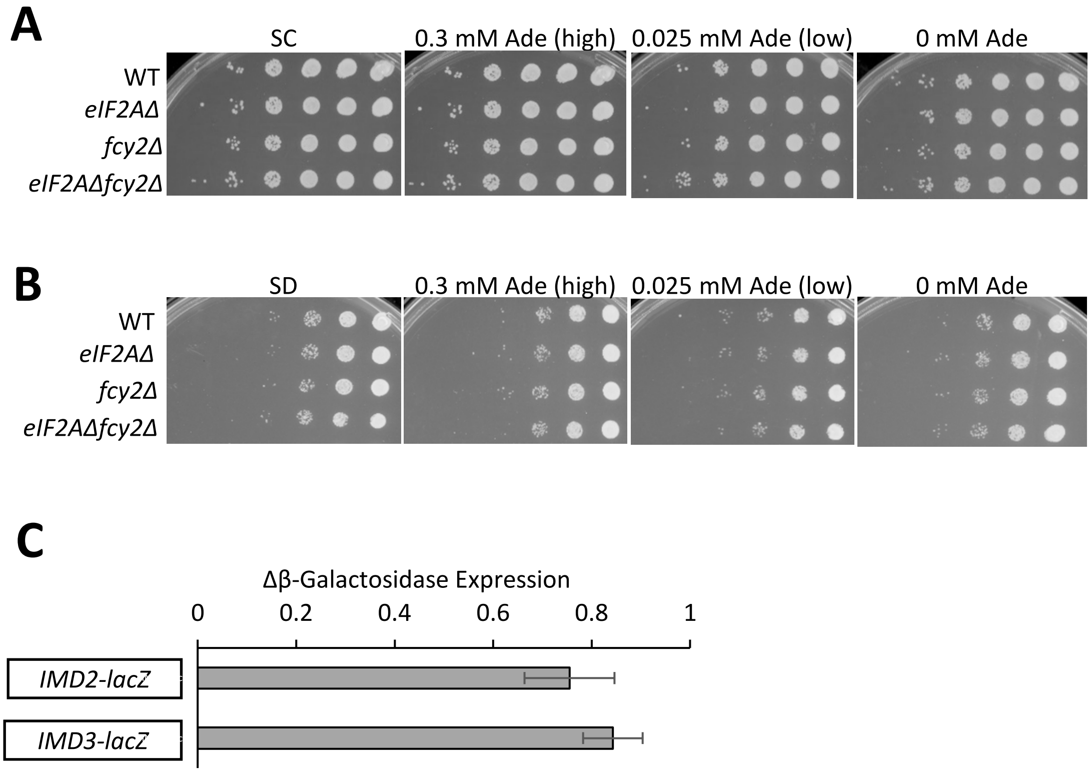

Figure 6-figure supplement 2

(which was not certified by peer review) is the author/funder. This article is a US Government work. It is not subject to copyright under 17 U
105 and is also made available for use under a CC0 license.

Figure 6-figure supplement 2. Lack of genetic interaction between eIF2A and the purine salvage pathway.

(A-B) Cell spotting assays were performed on SC plates (A) or SD (B) plates to assess the growth of WT, *eIF2AΔ*, *fcy2Δ* and *eIF2AΔ fcy2Δ* strains. Ten-fold serial dilutions of saturated cultures were applied to SC or SD plates supplemented with the indicated concentrations of adenine and incubated at 30°C for 2 days. **(C)** WT and *eIF2AΔ* strains were transformed with the indicated *lacZ* reporter plasmids- IMD2 and IMD3. The transformants bearing the reporter plasmids-IMD2 containing IMD2 coding sequences along with 1186 bp upstream region while IMD3 contains IMD3 coding sequences containing 555 bp upstream region- were grown in SC-Ura to saturation. The cultures were then diluted in fresh SC-Ura containing 0.015 mM concentration of Adenine and grown for 6 h to A_{600} of ~1.0. WCEs were prepared and assayed for β-galactosidase activities in units of nmol of ONPG cleaved per mg of protein per min. The results represent the fold change of means and ±SEMs of activities calculated from three independent transformants.
