# Supplementary material for: Yeast eIF2A has a minimal role in translation initiation and uORF-mediated translational control in vivo
Source: bioRxiv. 2023 Oct 8:2023.10.06.561292. Preprint. [Version 1] doi: 10.1101/2023.10.06.561292 (PMC10659434; doi:10.1101/2023.10.06.561292)
Supplement: Supplementary file 1 [file NIHPP2023.10.06.561292V1-supplement.pdf]

(which was not certified by peer review) is the author/funder. This article is a US Government work. It is not subject to copyright under 17 U
105 and is also made available for use under a CC0 license.

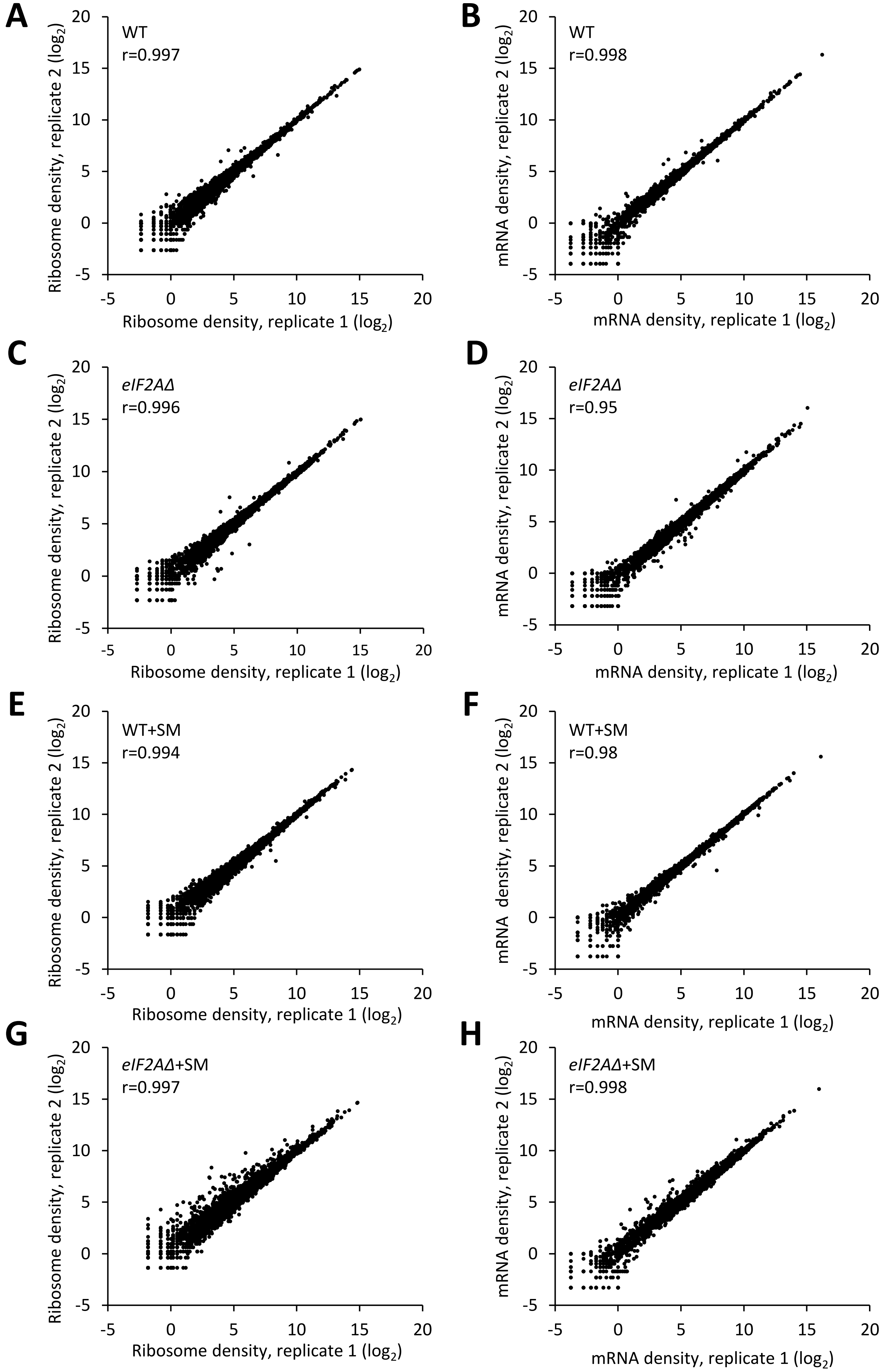

Figure 1-figure supplement 1

(which was not certified by peer review) is the author/funder. This article is a US Government work. It is not subject to copyright under 17 U
105 and is also made available for use under a CC0 license.

Figure 1-figure supplement 1. High reproducibility between biological replicates of ribosome footprint profiling and RNA-seq analyses.

(A-H) Scatterplots depict the RPF (A, C, E, G) or mRNA (B, D, F, H) read densities for all expressed mRNAs across biological replicates of the WT (A, B), the *eIF2AΔ* mutant (C, D) SM-treated WT (E, F) and SM-treated *eIF2AΔ* mutant (G, H). The read densities were calculated by mapping the reads to the CDS of each gene and expressed as reads per million mapped reads (RPM) in individual libraries of biological replicates. The Pearson's coefficient (r) is indicated in each plot, quantifying the degree of correlation between the replicate datasets.

(which was not certified by peer review) is the author/funder. This article is a US Government work. It is not subject to copyright under 17 U
105 and is also made available for use under a CC0 license.

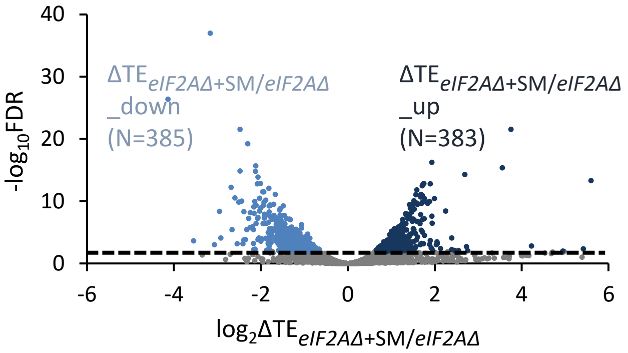

Figure 2-figure supplement 1. Relative TE changes evoked by increased eIF2α phosphorylation in cells lacking eIF2A.

Volcano plot as in Figure 2A showing the $\log_2$ ratios of TEs in SM-treated *eIF2A*Δ versus untreated *eIF2AΔ* cells ($\Delta TE_{eIF2A\Delta+SM/eIF2A\Delta}$ values) for the 5426 mRNAs with evidence of translation. The dotted line marks the 1% FDR threshold. Genes showing a significant increase ($\Delta TE_{eIF2A\Delta+SM/eIF2A\Delta}$_up) or decrease ($\Delta TE_{eIF2A\Delta+SM/eIF2A\Delta}$_down) in TE in SM-treated *eIF2AΔ* versus *eIF2AΔ* mutant cells at FDR < 0.05, are plotted in dark and light blue circles, respectively.

Figure 2-figure supplement 1

(which was not certified by peer review) is the author/funder. This article is a US Government work. It is not subject to copyright under 17 U
105 and is also made available for use under a CC0 license.

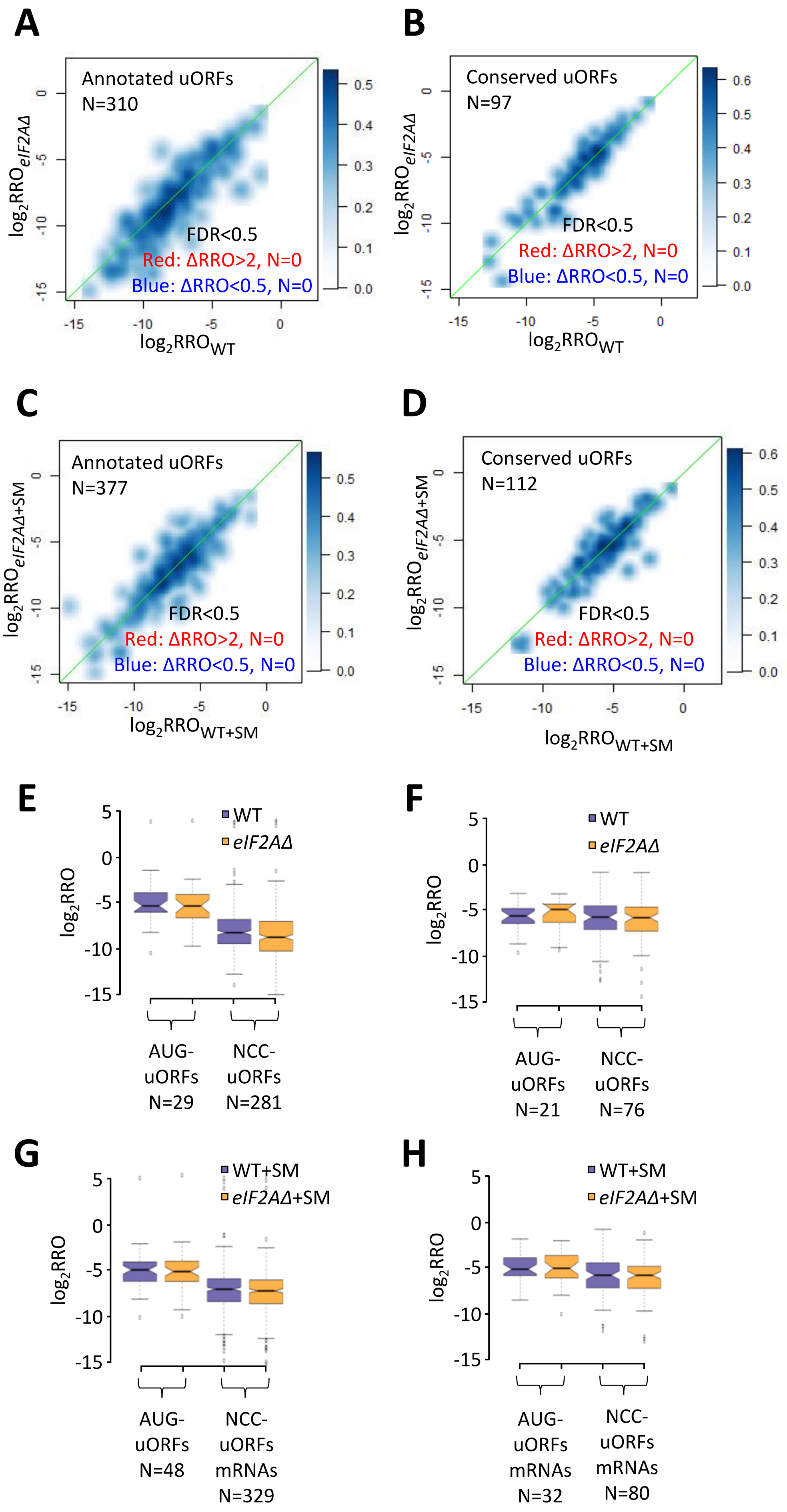

Figure 6-figure supplement 1

(which was not certified by peer review) is the author/funder. This article is a US Government work. It is not subject to copyright under 17 U
105 and is also made available for use under a CC0 license.

Figure 6-figure supplement 1. eIF2A plays a minimal in regulating uORF-mediated translation.

(A-B) Smoothed scatterplots displaying the relationship between log_2RRO_{WT} (x-axis) and $log_2RRO_{eIF2A\Delta}$ (y-axis) for all mRNAs containing annotated AUG- or NCC-uORFs (A) or evolutionarily conserved AUG- or NCC-uORFs (B) in WT versus *eIF2AΔ* cells without SM treatment. No mRNAs showed ≥2-fold changes in RRO in the *eIF2AΔ* mutant versus WT cells at FDR < 0.5. **(C-D)** Smoothed scatterplots displaying the relationship between log_2RRO_{WT+SM} (x-axis) versus $log_2RRO_{eIF2A\Delta+SM}$ (y-axis) for the same mRNAs analyzed in (A)-(B) but in the presence of SM. Again, no mRNAs showed ≥2-fold changes in RRO in the *eIF2AΔ* mutant versus WT cells at FDR < 0.5. **(E-H)** Notched box plot displaying log_2RRO values for all mRNAs containing annotated AUG- or NCC-uORFs (E, G) or evolutionarily conserved AUG- or NCC-uORFs (F, H) in untreated WT and *eIF2AΔ* mutant (E-F) or SM-treated WT and *eIF2AΔ* mutant (G-H). The y-axis scale was expanded by omitting a few outliers.

(which was not certified by peer review) is the author/funder. This article is a US Government work. It is not subject to copyright under 17 U
105 and is also made available for use under a CC0 license.

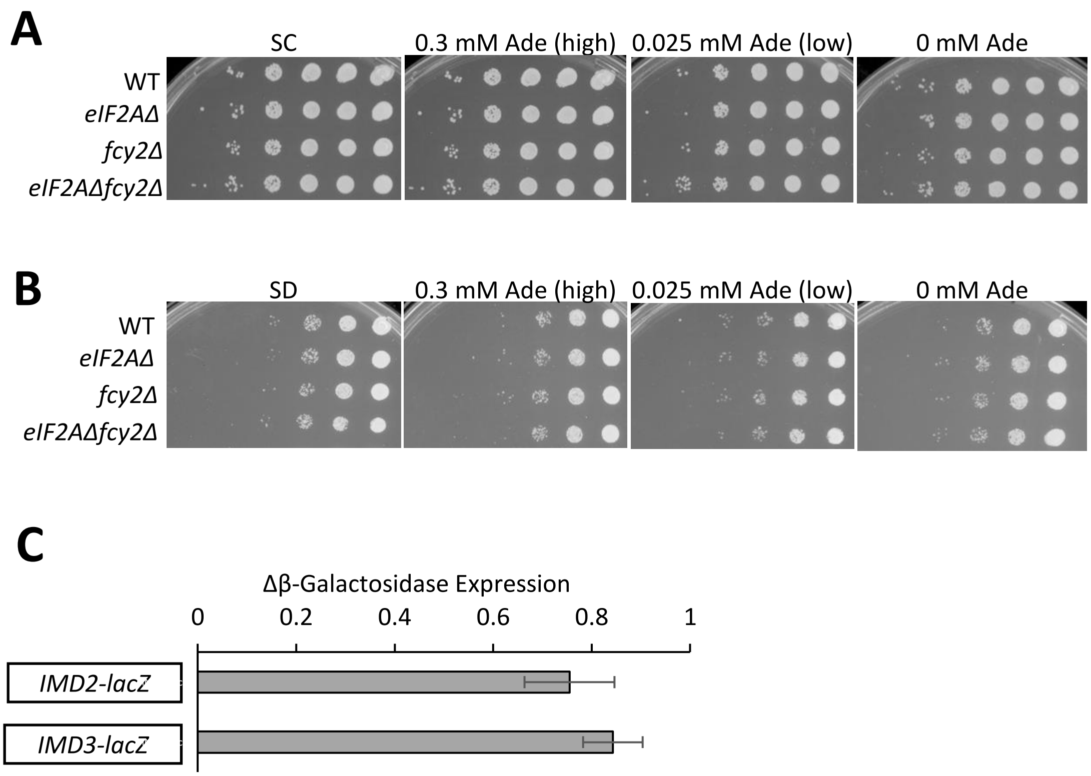

Figure 6-figure supplement 2

(which was not certified by peer review) is the author/funder. This article is a US Government work. It is not subject to copyright under 17 U
105 and is also made available for use under a CC0 license.

Figure 6-figure supplement 2. Lack of genetic interaction between eIF2A and the purine salvage pathway.

(A-B) Cell spotting assays were performed on SC plates (A) or SD (B) plates to assess the growth of WT, *eIF2AΔ*, *fcy2Δ* and *eIF2AΔ fcy2Δ* strains. Ten-fold serial dilutions of saturated cultures were applied to SC or SD plates supplemented with the indicated concentrations of adenine and incubated at 30°C for 2 days. **(C)** WT and *eIF2AΔ* strains were transformed with the indicated *lacZ* reporter plasmids- IMD2 and IMD3. The transformants bearing the reporter plasmids-IMD2 containing IMD2 coding sequences along with 1186 bp upstream region while IMD3 contains IMD3 coding sequences containing 555 bp upstream region- were grown in SC-Ura to saturation. The cultures were then diluted in fresh SC-Ura containing 0.015 mM concentration of Adenine and grown for 6 h to A_{600} of ~1.0. WCEs were prepared and assayed for β-galactosidase activities in units of nmol of ONPG cleaved per mg of protein per min. The results represent the fold change of means and ±SEMs of activities calculated from three independent transformants.
